# Cellular Regulation of the Uterine Microenvironment That Enables Embryo Implantation

**DOI:** 10.3389/fimmu.2015.00321

**Published:** 2015-06-17

**Authors:** Ana Claudia Zenclussen, Günter J. Hämmerling

**Affiliations:** ^1^Experimental Obstetrics and Gynecology, Medical Faculty, Otto-von-Guericke University, Magdeburg, Germany; ^2^Molecular Immunology, German Cancer Research Center (DKFZ), Heidelberg, Germany

**Keywords:** uterine environment, Treg cells, mast cells, uterine NK cells, macrophages, uterine DCs, neutrophils, implantation

## Abstract

Implantation of the fertilized egg into the maternal uterus is a crucial step in pregnancy establishment. Increasing evidence suggests that its success depends on various cell types of the innate immune system and on the fine balance between inflammatory and anti-inflammatory processes. In addition, it has recently been established that regulatory T cells play a superordinate role in dictating the quality of uterine environment required for successful pregnancy. Here, we discuss the cellular regulation of uterine receptivity with emphasis on the function and regulation of cells from the innate and adaptive immune system.

## Introduction

Pregnancy begins with the fertilization of the ovum, followed by implantation of the blastocyst in the maternal uterus. To implant, the blastocyst needs to aggressively adhere to the endometrium so that it can be provided with oxygen and nutrients. During this process, uterine tissue remodeling and inflammatory processes are required. After successful implantation, specific tolerance toward foreign paternal fetal antigens needs to be established (1) without loss of the ability to fight infections.

Much effort has been invested into the identification of the tolerance pathways responsible for smooth development of pregnancy. Maternal CD4+CD25+ Foxp3+ regulatory T cells (Tregs) are the most studied cells in this regard. They have been reported to contribute to the maintenance of tolerance during pregnancy by suppressing maternal alloreactive immune responses against paternal antigens in fetal cells (2–5). However, recent data showed that their participation at initial stages of pregnancy, e.g., implantation, may be much more relevant than at later stages when alloreactivity against the fetus needs to be prevented. Animals depleted of Foxp3+ Tregs presented impaired implantation in both, allogeneic and syngeneic matings, and their uterine characteristics reminded those of animals that were infected with Chlamydia and thus exhibited limited success of pregnancy (6). These observations lead us to propose that there is an important albeit unspecific role for cells of the adaptive immune system even at the time of implantation, when inflammatory processes and innate cell populations reportedly play an important role. When Tregs are deleted at later time points, pregnancy is much less jeopardized as some fetuses survive the temporary Treg absence [(3, 4) and unpublished data from Teles and Zenclussen]. Similarly, infertile patients have critically diminished Foxp3 mRNA levels in endometrium, supporting the concept of Foxp3+ Treg importance for pregnancy establishment (7). In our own studies, we observed that Foxp3+ Tregs are essential for implantation via modulation of the uterine microenvironment. In their absence, inflammation and fibrosis occur and blastocysts fail to implant. However, Tregs are not the only cells of relevance for implantation. In this review, we will dissect data available on different cell populations of both the innate and the adaptive immune system, and discuss the complex cellular and molecular network in the uterine microenvironment that favors implantation. We will address both mouse and human data, as it is important to learn whether data obtained in complex animal systems are relevant for the human beings. In view of the relevance of the topic for couples with infertility or subfertility, we will further discuss potential ways to positively modulate the uterine environment, thereby allowing a better implantation rate. In particular, data obtained in mice emerge as very informative for human pregnancies, which is most probably due to the fact that both species present hemochorionic placentation (8), and to the similarities of their immune systems.

KEY CONCEPTS

(1) A perfectly coordinated interplay between immune cells, hormones, and cytokines is crucial during peri-implantation. This can be greatly affected by environmental factors.
(2) Cells of the innate immune system are present at the uterus at fecundation and their presence is fundamental to initiate tissue remodeling that ensures implantation and later to support the adaptation of maternal spiral arteries.
(3) From the adaptive immune system, regulatory T cells emerge as critical for early pregnancy establishment. Their absence provokes inflammation and fibrosis in the uterus, which hampers implantation.
(4) Perturbations in the uterus, e.g., by removal of a particular cell population when using a mouse model, greatly affect the establishment of pregnancy. This is clinical relevant and goes in hand with observations done in infertile patients.

## Uterine Microenvironment during Peri-Implantation

During the period of periconception, the blastocyst is formed while the uterus prepares for implantation. For this to occur, a finely orchestrated balance of cells and soluble mediators is necessary. While genetic factors are relevant for the quality and viability of the blastocyst, the immune and endocrine systems are responsible for the formation of a perfect environment where the blastocyst, and later the embryo and fetus, will grow and develop into a healthy baby. For implantation to occur, it is necessary to have a receptive endometrium, a so-called healthy uterine milieu that allows the invasion of the blastocyst and the rapid growth of the placenta while supporting the transformation of uterine into decidual cells. This is facilitated by immune cell populations, the cytokines they secrete and, importantly, by hormonal changes. The immune cells that are relevant for implantation are present already before pregnancy. Immune cells that are present in the uterus usually display a unique, uterine phenotype that differs from the phenotype of their counterparts located either in the periphery or in other tissues. The unique phenotypes and possibly unique functional properties are likely provoked by the particular environment conditions that modify the cells and make them sui generis for a particular tissue. The most intensively studied uterine immune cells are the uterine natural killer cells (uNK cells) (9). Uterine dendritic cells (uDCs) also show a unique phenotype as well as demonstrated in both human beings and mice (10, 11). They are present in high numbers at specific sites along the non-pregnant uterus as shown *in vivo* (12). The same is true for uterine mast cells (uMCs) that differ enormously from MCs found in other tissues (13, 14), and for uterine macrophages that are crucial in modulating later trophoblast function (15). Some immune cells present in the uterus fluctuate in number through the different phases of the menstrual cycle in human beings or the estrus cycle in mice. This is true for uNK cells (16), macrophages (17), and Treg (6). These oscillations are probably mediated by hormonal changes (18). Other cells are likely to be attracted from the periphery to the uterus upon hormonal changes in early pregnancy as shown for uMCs (19) and macrophages (17). For other cells, e.g., uDCs, it is still unclear whether they reside in the uterus or are attracted by pregnancy hormones or both. From our *in vivo* microscopy data, uDCs are likely localized in the uterus at strategic niches that may indicate the future sites of implantation (12).

Immediately after copulation, the presence of seminal fluid attracts innate and adaptive immune cells due to the fact that seminal fluid can rapidly activate cytokine secretion (20). This is true for several animal species and has been most studied in mouse models. In the human cervix, Sharkey et al. (21) could show that the presence of seminal fluid after coitus attracts macrophages, dendritic cells (DCs), and T cells (21). In animal models, the specific depletion of macrophages, DCs, and mast cells (MCs) impairs implantation (13, 22, 23). Likewise, specific depletion of Tregs before implantation negatively affects the uterine environment and hinders implantation (6). Interestingly, human infertile women have very low levels of endometrial Foxp3, supporting the relevance of Tregs for implantation (7). Thus, the presence of crucial cells and the influx of new ones at peri-implantation dictate its success. This is, in turn, greatly affected by environmental factors such as nutritional state and medication. Excessive lipid storage in obese mice causes disorders of ovarian function that leads to follicular atresia (24). In addition, diet-induced obesity negatively affects uterine cells populations such as uNK cells (25). In a recent study, Bellver and colleagues reported that obesity in women impairs the reproductive outcome of ovum donation because of reduced uterine receptivity (26). A possible explanation for poor uterine receptivity in obese patients may be an aberrant Adipoq signaling in decidual cells leading to suboptimal decidualization (27). Hence, a perfectly coordinated interplay between immune cells, hormones, and cytokines is crucial during peri-implantation, and this can be greatly affected by environmental factors.

## Cells of the Innate Immune System in the Uterus and Their Relevance for Implantation

In general, cells of the innate immune system are the first to encounter microbes and foreign antigens; they recruit other cells by releasing chemokines and cytokines, and they activate the adaptive immune system so that an antigen-specific immune response can be initiated. In addition to acting as the first line of defense, cells of the innate immune system that are present in the uterus have an additional role. They actively contribute to pregnancy establishment either by modulating tissue remodeling or by releasing angiogenic factors that contribute to spiral artery (SA) remodeling. In addition, their activation status and cytokines dictate the uterine environment.

## Macrophages

Macrophages are abundant in the uterus. The numbers fluctuate during the estrus cycle (28), which is driven by estrogen and progesterone as shown in ovariectomized mice (17). Immediately after copulation, more macrophages are attracted to the endometrium by seminal fluid (29). Macrophages are present in tissues from early pregnancy losses but this is most probably a consequence and not a cause for pregnancy failure (30). Decidual macrophages are activated (31) and therefore able to present antigens to T cells. They have a cytokine profile resembling M2 macrophages that are programed into a immunosuppressive phenotype and secrete transforming growth factor (TGF)-beta and interleukin-10 [IL-10 (32, 33)]. They further produce other tolerance-related molecules such as indoleamine 2,3 dioxygenase [IDO (34)]. Macrophages were reported to modulate trophoblast function *in vitro*. Fest et al. showed that trophoblasts actively recruit monocytes to produce cytokines that in turn will support their own survival (15). Leukemia inhibitory factor (LIF) deficient mice are infertile because of implantation failure, but pregnancy can be rescued by LIF administration at day 4 of pregnancy (35). Lif *knockout* mice that were mated were investigated for their cell populations at early pregnancy without LIF application in order to delineate the cellular changes that are associated with failed implantation. Macrophages were reduced in numbers by more than half in LIF knockout vs. wild-type mice (36). This observation indicates a role for macrophages in implantation. More evidence for the importance of macrophages was recently provided by Sarah Robertson’s group. Care and colleagues reported that specific depletion of CD11b macrophages resulted in implantation failure, owing to the fact that macrophage depletion altered the luteal microvascular network that is necessary for the integrity of the corpus luteum and progesterone production (22). This finding may explain the subfertility of women with luteal insufficiency. Recently, the location and density of macrophages in human implantation specimens was studied *in vitro*. At first trimester placentation sites, a higher density of decidual macrophages was found in close proximity to the invasive trophoblast (37), which supports the pioneering findings from Fest et al. (15), who reported that the interaction with trophoblasts modifies the phenotype of macrophages. Indeed, decidual macrophages were postulated to contribute to trophoblast invasion and placenta development (34). In pregnancy pathologies, levels of macrophage-associated molecules are reportedly diminished, e.g., granulocyte-macrophage colony-stimulating factor [GMCSF (38)]. In human beings, CD163^high^ endometrial macrophages constitutively secrete both pro- and anti-inflammatory cytokines and pro-angiogenic factors (39). Recently, Siwetz et al. reported that the addition of fractalkine significantly impaired the ability of monocytes to adhere to trophoblasts (40). Fractalkine is upregulated in pregnancy pathologies such as chorioamnitis (41) suggesting that in pregnancy pathologies, the communication between trophoblasts and monocytes may be disturbed. These observations point also to the relevance of macrophages inhuman pregnancies.

## Neutrophils

Neutrophils are short-lived and function as effector cells. In general, they do not reside in tissues but are recruited from the circulation to the sites where they are needed, usually to sites of inflammation or infection (42). Tissue infiltrating neutrophils can have angiogenic properties and have a role in the late stages of tumor progression through enhancement of angiogenesis and vascular remodeling (43). This is similar to what is known about M2 tumor-associated macrophages (44). Recently, two tumor-associated neutrophil populations were identified: N1 antitumorigenic neutrophils, with cytotoxic and immune stimulatory potential; and N2 protumorigenic neutrophils, with immunosuppressive and angiogenic properties, but lacking cytotoxic potential (45). Interestingly, tissue resident neutrophils in non-pregnant human fallopian tubes are less cytotoxic than peripheral blood neutrophils (PMNs) and exhibit higher cytokine production, particularly of vascular endothelial growth factor (VEGF) (46). Amsalem and colleagues recently described a novel population of second-trimester decidual neutrophils (dNs) (47). Under the influence of the decidual microenvironment, mainly of decidual CXC-motiv-chemokine (CXCL8), these cells adopt a unique phenotype that is different from the phenotype of PMNs, similar to dNK cells, decidual macrophages, and uNK cells. The data suggest that this population displays angiogenic properties. The authors also observed the presence of this population in mice. In both human and mice samples, these cells were spatially restricted to the decidua basalis (DB). When compared to PMNs, the dNs expressed high levels of neutrophil activation markers and angiogenesis-related proteins such as VEGF, Arginase-1 (ARG-1), and chemokine ligand (CCL)2 (47). Functional *in vitro* assays showed that supernatants from second-trimester human decidua stimulated transendothelial PMN invasion, upregulated VEGF, ARG1, CCL2, and intercellular adhesion molecule (ICAM)1 mRNA levels, and increased PMN-driven *in vitro* angiogenesis in a CXCL8-dependent manner (47).

Contrary to this positive function, recent studies by Mizugishi and collaborators suggest that excessive number of neutrophils is pregnancy deleterious. These authors showed that the disruption of the sphingosine kinase gene (Sphk) during pregnancy causes an upregulation of CXCL1 and CXCL2 in the decidua that in turn provokes a massive influx of neutrophils into the feto-maternal interface with enhanced oxidative damage, resulting in early fetal death (48). Sphk-deficient mice exhibit neutrophilia in peripheral blood and a decrease in the number of decidual natural killer cells. The blockage of neutrophil influx protected Sphk-deficient mice against pregnancy loss (48). Specific depletion of neutrophils in mouse models and functional studies with dNs will help in the future to understand their role in implantation and pregnancy.

## Dendritic Cells

An important function of DCs is the presentation of antigens to T cells. This step is also relevant in pregnancy as the nature of antigen presentation defines the fate and the quality of the immune response that will lead to tolerance or rejection of the blastocyst. Our data obtained in mouse models strongly suggest that the first encounter between male antigens present in the sperm and maternal DCs takes place in the vaginal lumen immediately after copulation (49). This is in agreement with a number of reports indicating that also in human beings seminal fluid containing male antigens attracts maternal immune cells such as DCs (21, 50). In mice, DCs are also located in the endometrial tissue when the female is sexually receptive (51). We could observe that DCs are very abundant in murine uterus during estrus, thus at sexual receptivity (12). Because only low numbers of DCs could be isolated from the uterus, it was believed that they were present only in low density. However, by employing *in vivo* microscopy, we could demonstrate that DCs are abundant in the uterus. Moreover, uDCs are located in cluster-like structures along the uterus may indicate the future sites of implantation (12). Like uterine macrophages, neutrophils, dNKs, and uMCs, uDCs diplay a distinctive phenotype compared with circulating DCs or DCs resident in other tissues. DC-SIGN + CD14 + CD83−DCs found in the uterus represent a unique subpopulation capable of activating inducible Tregs (52). We found heme oxygenase-1 (HO-1) and the pregnancy molecule human chorionic gonadotropin (hCG) to be important modulators of uDC maturation state, both implied in their maintenance as immature tolerogenic DCs (53, 54). GM-CSF is also an important regulator of DC maturity, and similar to hCG and HO-1, it confers DCs an immature phenotype (55). Recently, Du et al. (56) showed that DCs are involved in the communication between trophoblasts and decidual Tregs. Negishi et al. recently showed that the depletion of 33D1(+) DCs during the perinatal period caused substantial fetal loss mediated by IL-12. These findings show the importance of balancing DC subsets during pregnancy. Interestingly, progesterone could rescue the abortions, highlighting the role of hormones in the balance of the pregnancy immune response (57).

The importance of DCs for pregnancy establishment was also suggested by Plaks and colleagues who observed in CD11c.DTR mice that animals depleted of DCs were unable to implant and that this failure was related with their ability to modulate uterine receptivity. DCs emerge as important cells for uterine tissue remodeling and angiogenesis (23). Whether mice that are constitutively devoid of DCs (58) have impaired fertility or suboptimal pregnancies has not been studied. So far, it seems that depleting DCs disrupts uterine integrity and this interferes with implantation. In human beings, DCs of an immature, and thus tolerogenic phenotype, are present and abundant in the endometrium and seem not to fluctuate throughout the menstrual cycle (59). They are also present in the decidua of early pregnant women (60). Human decidual DCs from early pregnancy express the tolerogenic markers IL-10, human leukocyte antigen (HLA)-G, and leukocyte immunoglobulin-like receptor subfamily B member 2 [LILRB2; (61)]. It was observed that the proportion of the described decidual DC-SIGN + cells (10) was significantly lower in samples from miscarriages when compared to samples from women with normal pregnancies (62). As in mice, it seems that the balancing of DC subsets is relevant for pregnancy maintenance and that several molecules, most prominently cytokines and hormones, are involved in keeping this balance.

## Uterine Mast Cells

Mast cells are present in the female reproductive tract (63–65). Until recently, their contribution to pregnancy establishment and/or maintenance was unclear as contradictory reports could be found in the literature. In pregnant rats, MC degranulation was shown to have positive effects on cervical angiogenesis (66). In mice, Menzies et al. found no role for these cells in labor in a syngeneic context when employing C57BL/6J-*Kit^W-sh/W-sh^* mice (67). We recently reported that, in adult female mice, a transient population of MCs appears cyclically within the uterine endometrium (13). As described for other cells of the innate immune system, uMCs represent a different population of MCs compared to MCs found in other tissues. uMCs are a mixed population of connective tissue-type MCs, mucosal MCs, and a transitional type that share features of both MC types. They express CD117 and FcεRIα, while only a small population expresses Mcpt5 and Mcpt8 (13). uMCs peak in number at the fertile phase of the murine estrous cycle (13). They remain high in numbers if pregnancy establishes. By using *in vivo* microscopy, we could confirm that uMCs are present in abundance in the uterus of pregnant C57BL/6J Mcpt5-Cre ROSA26-EYFP mice (14). They are localized in close proximity to blood vessels (13, 14). This is likely regulated by endocrine mechanisms. Indeed, estradiol is able to potentiate MC degranulation *in vitro* (68). We recently found that estradiol and progesterone promote MC migration from the periphery to the uterus of ovariectomized mice and favor subsequent maturation of these cells *in situ* (19). *Kit^W-sh/W-sh^* mice lacking MCs were described as “fertile” (69); however, *Kit^W-sh/W-sh^* colonies often show irregular birth rates and high natal and postnatal death rates. We observed that in allogeneic, clinically relevant pregnancies, MC-devoid *Kit^W-sh/W-sh^* female mice displayed severely impaired implantation, although single females presented normal litter sizes (13). The transfer of wild-type bone marrow-derived MCs (BMMCs) was able to completely rescue the phenotype (13). These findings support the positive role of MCs in normal implantation. In our model, transferred MCs migrated to the fetal–maternal interface and the local injection of these cells could also recapitulate a normal phenotype (13). Hence, MCs act locally within the uterus to foster normal pregnancy. MC-chymases were present after adoptive or local BMMCs transfer. Because chymases contribute to matrix degradation and tissue remodeling and induce sprouting of new blood vessels (70), we hypothesized that MCs are important initiators of tissue remodeling during pregnancy. Indeed, other studies in rat showed that the pro-angiogenic process that takes place in the uterine cervix during rat implantation is regulated by the pro-angiogenic factor VEGF-A released by MCs upon degranulation (66, 71). Besides fostering implantation, we found that uMCs are involved in SA remodeling in later pregnancy (13). MC-deficient mice presented abnormally remodeled SA and the transfer of BMMCs could revert this phenotype (13). The glycan-binding protein galectin-1 (Gal-1) seems to participate in this process as the transfer of *Lgals1^−/−^* BMMCs was unable to normalize pregnancy outcome (13). Thus, uMCs are important cellular players that support tissue remodeling during implantation, and later positively influence angiogenesis (66), placentation, and fetal growth (13). Hence, like other cells of the innate immune system, MCs that are present in the uterus are phenotypically different from MCs residing in other tissues and contribute to pregnancy establishment. Whether or not this finding reflects also the situation in pregnant women is unknown.

## Uterine Natural Killer Cells

Uterine natural killer cells represent the major immune cell population at the early feto-maternal interface; they are key regulators of the maternal uterine vasculature remodeling (72). As for other innate immune cells, uNK cells greatly differ in phenotype and function from peripheral NK cells. Similar to uMCs, they are also localized close to vessels in the DB and the mesometrial lymphoid aggregate of pregnancy (MLAp) (73, 74). CD3^−^CD122^+^ uNK cells do not express the common NK markers NK1.1 and DX5, but bind selectively and specifically to the lectin *Dolichos biflorus* agglutin (DBA) (75). Besides production of pro-angiogenic factors VEGF (76), placental growth factor (PGF) (77), and Delta-like ligand (78), mouse uNK cells secrete local interferon (IFN)-γ that is necessary and sufficient for the initiation of SA remodeling (79). Mice devoid of uNK cells, like IL-15 KO mice that do not have NKs or uNK cells, have impaired SA modification and placenta development, resulting in intrauterine growth restriction (IUGR) and smaller progeny (80), but it has to be kept in mind that in addition to NK deficiency IL-15 KO mice display other impairments, which may affect pregnancy. In human beings, uNK numbers fluctuate during the menstrual cycle (16) but in mice they are present in comparable numbers throughout the estrous cycle and expand only if pregnancy takes place (81). We found that HO-1 and its metabolite carbon monoxide (CO) are important modulators of uNK cell number. HO-1 deficient animals show a significant reduction in the absolute number of uNK cells as early as gestation day 8 (82); these findings are in agreement with observations by Zhao et al (83). We postulated that the absence of HO-1 interferes with the phenotype of macrophages (M1 rather than M2) that in turns provokes less IL-15 secretion, thereby reducing uNK cell numbers (81). HO-1 deficiency resulted not only in fewer uNK cells but also in shallow SA remodeling and IUGR (82). The administration of CO from gestation day 3 until day 8 provoked a significant increase in the number of uNK cells at the feto-maternal interface that was independent of IL-15 (82). CO treatment rather provoked the *in situ* proliferation of uNK cells (82). Furthermore, CO application to pregnant *Hmox1*^+∕−^ females enhanced the expression of VEGF, PGF, and IFN-γ that accompanied SA reshaping. In a more clinically relevant aspect, it was shown that CO application during early pregnancy can actually prevent hypertension and IUGR in HO-1-deficient mice (82). The importance of the microenvironment at early pregnancy and the relevance of immune cells, in particular of uNK cells, are highlighted by these experiments. In HO-1-deficient animals, the rise in the blood pressure began at day 14 while the CO treatment that could prevent it was performed between days 3 and 8 of pregnancy. Hence, the events leading to gestational hypertension are triggered and can be corrected beginning as early as day 3 in the mouse. One important hallmark of the blood pressure normalization is the expansion of uNK cells and changes in angiogenesis at the feto-maternal interface. In human beings, a shallow invasion of the trophoblast at week 14, long before pre-eclampsia symptoms arise in patients, is responsible for the pathophysiology of this complex disease (84). These observations illuminate the relevance of a balanced microenvironment and the importance of specialized cells of the innate immune system. Contradictory data can be found in the literature regarding uNK cell function in human beings, and more studies are needed to elucidate their function and regulation.

## Cells of the Adaptive Immune System in Early Pregnancy Success

Contrary to the original hypothesis from Peter Medawar in the 1950s, it is known that the maternal immune system is not ignorant but aware of the growing conceptus. In this context, it is important to note that trophoblasts have reduced antigenicity and attenuated expression of molecular histocompatibility (MHC) genes, but transplantation antigens are clearly expressed (85). Moreover, owing to the bidirectional cell trafficking between mother and fetus antigen presentation and recognition can take place anywhere in the maternal immune system. This recognition needs to induce tolerance mechanisms so that the fetus is tolerated and not rejected by classical immune mechanisms. We found that paternal antigens are present in several immune and non-immune maternal organs during the whole period of pregnancy. The first place of encounter of paternal antigens is the vaginal lumen, where maternal DCs meet paternal antigens present in seminal fluid (6, 49). Antigens present in sperm and in particular in seminal fluid are sufficient to elicit an expansion of a particular T cell type: Tregs. Females mated with vasectomized males present levels of Tregs comparable with females mated with intact male; the mating of females with males whose seminal vesicles had been previously removed was associated with a lack of Treg expansion in the periphery and in paraaortic lymph nodes (6, 86). T cells that are reactive for paternal/fetal antigens are detectable in peripheral blood and decidua of healthy pregnant women (87). Mice with transgenic T cells that react to fetal antigens are present and functional since early pregnancy (55). Hence, maternal T cells are crucial in recognizing and tolerating the invading fetus (1). No information is available about the participation of B cells at the early stage blastocyst implantation. We have reported, however, that failure of IL-10 producing B cells to expand is related with miscarriages. *In vitro*, these cells were able to hamper tumor necrosis factor (TNF)-alpha production by effector T cells (88). In mice, early transfer of IL-10 producing B cells can rescue from immunological abortion (89). Whether this is related to events taking place in early pregnancy is unknown. Because B cell functionally can be modified by pregnancy hormones [reviewed in Ref. (90)], it is assumed that they are also implied in early pregnancy and future studies will reveal their importance.

## Regulatory T Cells

Much effort has been devoted to the understanding of the role of cells and molecules involved in generation and maintenance of tolerance that guarantees pregnancy success. Even though initial studies concentrated on T helper cells able to produce either Th1 or Th2 cytokines, it is now clear that a more specialized subset of T cells with regulatory function is the main regulator of specific immunosuppression. These cells are Tregs. Tregs are crucial players in the avoidance of autoimmunity, and their role in physiological and pathological processes has been studied since their discovery in 1995 (91). There is consensus that Tregs are relevant for pregnancy. Pioneering studies from Aluvihare and colleagues (92) and our own laboratory in 2005 (2) revealed their pivotal role for pregnancy success.

We have recently reported that, in vaginal lavage, Tregs peak at the receptive phase of the estrous cycle, namely estrus. *In vivo* 2-photon microscopy in *Foxp3^gfp^* animals impressively demonstrated a clustering of Tregs in uterine tissue during estrus that was not observed during the other phases of the oestrus cycle (6). These data can be interpreted as a preparation of the uterus for pregnancy and, therefore, show similarities with the cycle-dependent expansion of innate immune cells in the uterus (12, 13, 22). When compared to virgin mice, an expansion of Tregs was observed in the uterine draining lymph nodes from females paired with intact or vasectomized males, whereas no expansion was found in females mated with seminal vesicle-deficient males. These data demonstrate the proliferative effect of seminal fluid on Tregs *in vivo*. These results could be reproduced *in vitro* where we could additionally show that Treg proliferation is inhibited by anti-TGF β antibody (6). Thus, the uterine accumulation of Tregs at sexual receptivity is followed by an expansion triggered by seminal fluid, most likely by TGF-β contained herein. These observations are in accordance with data from Robertson’s group (93). Hence, Treg presence at the moment of pairing and their further expansion by paternal components imply a role in implantation. To further address these findings, Foxp3^+^ Tregs were depleted in *Foxp3^DTR^* (94) mice by application of human diphteria toxin (DT). Treg depletion beginning at d-9 in *Foxp3^DTR^* mice provoked a failure of the embryos to implant, whereas PBS- and DT-treated control mice displayed normal implantation (6). In a second set of experiments, Tregs were depleted in BAC transgenic Foxp3.LuciDTR mice (95) in syngeneic and allogeneic matings. Treg ablation from d-2 to d5 resulted in severely impaired implantation not only in biologically relevant allogeneic pregnancies but also in syngeneic matings (6). At this early time point, the most important function of Tregs is probably the prevention of pro-inflammatory events that may occur during implantation. Without Tregs, inflammation may be too strong and hinder the nidation of the embryo. Indeed, we observed that Treg depletion resulted in accumulation of uterine CD8^+^ T cells and of activated T cells in lymph nodes draining the uterus (6). Additionally, the uterine tissue was inflamed and thickened after Treg depletion treatment. The inflammatory mediators IL-15, IL-6, chemokine receptor (CCR)5, CXCL11, CCL19, and CXCL3 were upregulated, and fibrosis could be detected in mice devoid of Tregs (6). Thus, depletion of Tregs prior to mating leads to a hostile uterine environment for implantation to take place, which is characterized by the occurrence of inflammation and fibrosis. A similar scenario was observed in CCR7-deficient mice. The number of CD4^+^Foxp3^+^ Treg in the uterus was drastically reduced in these mice and, consistently, this resulted in implantation failure (6). Thus, CCR7 mediates the homing of Treg to the uterus and its absence hampers implantation. Interestingly, Guerin et al. reported the presence of CCL19, the ligand for CCR7, in glandular and luminal uterine epithelial cells (93). In mice, depletion of Tregs or absence of Tregs in the uterus, as in CCR7-deficient females, resulted in a dramatic impairment of implantation. It can be speculated that CCR7/CCL19 are implied in the migration of thymic Tregs to the uterus (as it is known for other tissues) and that pregnancy-specific molecules support Treg migration and/or expansion once fertilization takes place. Treg presence before implantation emerges as important for the generation of an adequate microenvironment so that the blastocyst can attach and implant. In infertile women, Foxp3 is diminished in endometrium (7). This would support our hypothesis. In mice that were specifically depleted from Tregs before pregnancy, we observed swollen uteruses that presented no viable implantations. Pelvic inflammation, uterine swelling, and intraluminal occluding fibrosis of the oviduct after infections with Chlamydia sp. are often associated with infertility (96, 97). It seems that without Tregs, inflammation overcomes and favors a rather hostile uterine microenvironment that hinders the nidation of the embryo. The genetic ablation of HO-1, which leads to inflammation too, is leading to failing implantation as well (98). Thus, Tregs are pivotal for implantation as they positively modulate the uterine environment that is needed for nidation. In their absence, the communication between the uterine tissue and the blastocysts is impaired, hindering the proper implantation of the latter.

Contrary to previous assumptions, it seems that Tregs are relevant for establishing rather than maintaining pregnancy. In our own studies, depletion of Tregs during mid-pregnancy did not interfere with its course (unpublished data). In another study, Samstein et al. (4) employed the same Foxp3^DTR^ knock in mice used by us (94), in which DT application depletes more than 95% Tregs, and reported a modest resorption rate of 10% when Tregs were ablated at d5.5 after the establishment of pregnancy. Thus, pregnancy is not seriously jeopardized when Tregs are depleted once implantation has taken place, although Tregs are absolutely required for pregnancy to establish. However, there are also conflicting reports in the literature. It was reported that Treg ablation with anti-CD25 antibody at day 4.5 post conceptum would result in a fetal absorption rate of about 50% in allogeneic but not syngeneic matings, and that depletion at day 2.5 would prevent implantation, again only in allo but not syngeneic matings (3). Since Foxp3.DTR mice are more specific for Treg ablation and deplete a much more efficiently than CD25 antibody, the latter data may have to be regarded with caution.

Once pregnancy is established, additional and multiple mechanisms must exist to ensure maternal tolerance toward the fetus. Several distinct tolerance mechanisms have been reported, such as awareness of maternal T cells for paternal alloantigens and acquisition of a transient state of tolerance during pregnancy (1), ignorance of fetus-specific T cells, epigenetic silencing in decidual tissue of chemokines that attract T cells (99), and other mechanisms. Current studies concentrate on the pathways of activation and proliferation of Tregs during early pregnancy. The available studies agree that functional Tregs in sufficient numbers are needed during peri-implantation. Several authors observed expanded population of Tregs within the uterine lymph nodes and in the uterus as early as day 2 of pregnancy (86, 100, 101). In addition, the transfer of Tregs before implantation can rescue the fetuses from immunological rejection in the CBA/J × DBA/2J combination, while later transfer has no effect (2). Furthermore, it seems that already at this early time point, antigen-specific Tregs are needed; the adoptive transfer of Tregs isolated from non-pregnant or unrelated pregnant females have no protective effect (102). In the same line, it was showed that depletion of Tregs with an anti-CD25 antibody at the post-implantation period does not jeopardize pregnancy (3). Although many studies show a defective expansion of Treg in the periphery of women suffering from early abortions, it appears more relevant to study their abundance in the uterus in women that are able to get but not stay pregnant. Whether the frequency of uterine Tregs can be deduced from blood testing is yet not known and merits further studies.

## Conclusion and Open Questions

Cells of the innate immune system are normally present in the uterus. Some of them fluctuate in numbers as a consequence of hormonal changes at different phases of the estrous cycle in rodents and menstrual cycle in women. Depletion of DCs, macrophages, and the absence of MCs results in implantation failure. The studies outlined above suggest that they are involved in the preparation of the uterus for pregnancy and contribute to peri-implantation through secretion of cytokines, modulatory molecules, and angiogeneic factors. Moreover, these cells appear also to be also relevant for tissue remodeling and for supporting the correct shaping of spiral arteries. It is also tempting to speculate that they have multiple functions at different time points and that they adapt to the changes of the environment by secreting different mediators. As the immune system has evolved about 500 million years ago and is, therefore, much older than placentation, which dates back from about 150 million years ago, it is possible that maternal immune cells present in the pregnant uterus have adapted in order to mediate tissue homeostasis and to support implantation. Many of the concepts arisen from animal studies have to be interpreted with caution because they are based on depletion of the respective innate cell populations. For example, depletion of DCs in CD11c.DTR mice completely prevents implantation (23), at first sight indicating an indispensable role for DCs in pregnancy. No studies were performed to study fertility in two independently derived strains of CD11c.DTA mice that express the diphtheria toxin A chain under control of the CD11c promoter and therefore lack DCs from birth on (58, 103). The authors report that pups are apparently born at normal mendelian rates; however, this is no guarantee for optimal fertility or pregnancy. Subfertility can imply a longer time period needed until the female gets pregnant and suboptimal implantation or placentation can lead to IUGR with serious implications for fetal development. This cannot be predicted just by observing that female mothers give birth to pups in normal mendelian rates. In these mice that are constitutively devoid of DCs, a major effect as observed when depleting DCs (23) can be however excluded, otherwise there would be no living pups. It is evolutionarily highly unlikely that other cell types can compensate for the absence of DCs; however, in their absence, other cells may secrete the factors that DCs normally secrete and so guarantee the development of the optimal uterine environment for implantation. The apparent discrepancy to the conditional DC depletion in CD11c.DTR mice can probably be best explained by a perturbation of tissue and cytokine balance in the uterus caused by the sudden removal of DCs. Whether one single type of cell or a combination of cells, or the molecules they secrete are indispensable for pregnancy is not yet known. The special phenotype and functional characteristics of uNK cells argues for an important role of uNK cell. No studies are available with mice that lack exclusively uNK cells. The same is true for uMCs. The recently generated CreMaster mice, which are reportedly specifically deficient for MC cells (104) and also lack uMCs (unpublished observations) were also not studied for their fertility. However, independent of the question whether or not particular innate cells are indispensable for pregnancy, the clinically important aspect that emerges from these studies is that perturbations in the uterus, e.g., by removal of a particular cell population, greatly affect the establishment of pregnancy.

As for cells of the adaptive immune system, Tregs emerge as the relevant T cell subset for conditioning the uterus for the blastocyst to implant. Their depletion results in a hostile environment that impedes normal placentation. Since the outcome of pregnancy in T cell deficient RAG mutant mice is not as dramatic as it is if depleting Tregs before mating, it seems that effector T cells are the major targets and that uterine Tregs serve to prevent excessive activation of T cells and production of inflammatory cytokines that would lead to dangerous perturbation of uterine tissue homeostasis. Interestingly, infertile women present low levels of Tregs. Moreover, the uterine environment is negatively modified in women that suffer from subclinical infections, e.g., with Chlamydia sp., which may explain why they fail to become or stay pregnant. The histological modifications of the tissue specimens from mice infected with Chlamydia show the same features as those observed after Treg depletion, namely an inflamed tissue with signs of fibrosis. More clinical data are needed to understand whether changes in cells of the adaptive immune system are related with infertility.

**Figure 1:**
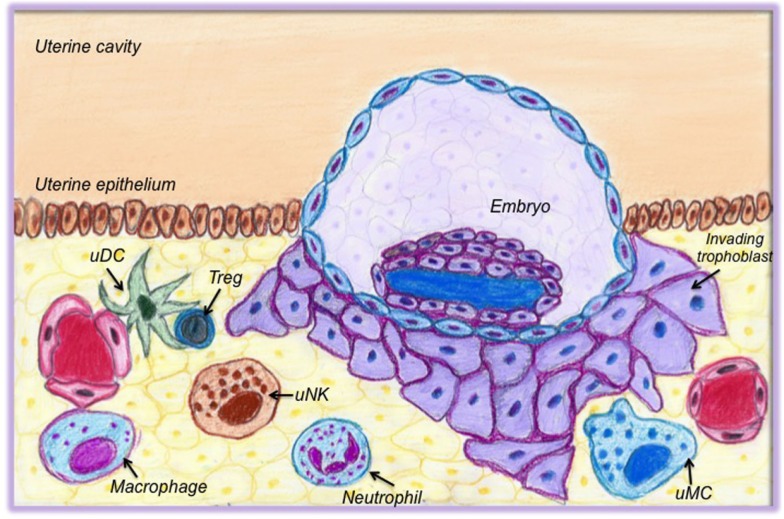
**Cells of the innate and adaptive immune system present in the uterus at the time of implantation**. This cartoon graphically depicts the abundance of immune cells and their close proximity with the invading trophoblast. Uterine DCs (uDCs) are localized close to vessels and in proximity to regulatory T cells (Treg). They act as mediators between Tregs and trophoblasts; they support angiogenesis and are responsible for early antigen presentation. Treg in turn are essential for preparing the uterus for implantation; they are responsible for early tolerance toward paternal antigens. Macrophages actively communicate with trophoblasts and support the luteal vascular network. Uterine natural killer cells (uNK cells) are important for tissue remodeling, to promote angiogenesis, and to contribute to the shaping of spiral arteries. Neutrophils and uterine mast cells (uMCs) both support angiogenesis. uMCs also modulate the remodeling of spiral arteries.

We have summarized the available information on the possible role of immune cells for early pregnancy in Table 1. In Figure 1, a cartoon intends to graphically depict the abundance of immune cells and their close proximity with the invading trophoblast.

**Table 1:** **Available information about the function of innate and adaptive immune cells at early pregnancy**.

Cell type	Role	Evidence	Reference
**INNATE IMMUNE SYSTEM**			
Macrophages	Modulation of the initial immune response	Antigen presentation at the feto-maternal interface, secretion of tolerogenic factors	Renaud et al. (34)
	Communication with trophoblasts	Trophoblasts actively recruit monocytes and modify their function	Fest et al. (15)
	Important for implantation	Specific macrophage depletion hinders implantation	Care et al. (22)
	Support luteal vascular network needed for progesterone production	Progesterone supplementation rescues the implantation phenotype of mice devoid of CD11b macrophages	Care et al. (22)
Neutrophils	Support of angiogenesis	Uterine neutrophils produce VEGF	Smith et al. (46)
		Second-trimester neutrophils secrete angiogeneic factors under the influence of decidial-like environment	Amsalem et al. (47)
Dendritic cells	Preparation of the uterus for implantation	DC presence and clustering during the sexual receptive phase	Zenclussen et al. (12)
	Early antigen presentation to T cells and Tregs	Presence of DCs in vaginal lumen attracted by seminal fluid	Zenclussen et al. (49) Sharkey et al. (21)
	Support decidual transformation	DC depletion impaired decidual proliferation and decidualization	Plaks et al. (23)
	Mediators of the communication between trophoblasts and Tregs	DCs that were activated by trophoblasts promote de conversion from naïve into Treg cells	Du et al. (56)
Uterine mast cells	Promote angiogenesis at the feto-maternal interface	MCs degranulation had positive effect on cervical angiogenesis	Bosquiazzo et al. (65)
	Support implantation	Uterine MCs secrete VEGF	Varayoud et al. (70)
	Positively influence tissue remodeling	MC-deficient ckit knockout mice have impaired implantation; reconstitution with BMMCs can correct this	Woidacki et al. (13)
	Modulate the remodeling of spiral arteries	Transfer of MCs into MC-deficient mice was related to increased TGFbeta/CtGF	Woidacki et al. (13)
	Support fetal growth	MC-deficient mice presented badly remodeled spiral arteries and this could be rescue by MCs	Woidacki et al. (13)
		MC-deficient animals presented smaller fetuses and this could be corrected but wild type but not Gal-1 deficient MC transfer	Woidacki et al. (13)
Uterine natural killer cells	Important for the remodeling of spiral arteries	Initiate the remodeling of spiral arteries, this is mediated by IFNgamma	Ashkar et al. (78)
	Promote angiogenesis at the feto-maternal interface	*In vivo*, CO treatment provoked the *in situ* expansion of uNKs and VEGF secretion	Greenwood et al. (80) Linzke et al. (81)
**ADAPTIVE IMMUNE CELLS**			
Regulatory T cells	Preparation of the uterus for pregnancy	Tregs accumulation at murine sexual receptivity in the uterus	Teles et al. (6)
	Early tolerance of paternal antigens expressed in the fetus	Upon attraction by seminal fluid, Treg are expanded by paternal antigens	Guerin et al. (92)
	Contribute to a friendly uterine environment that ensures implantation	Specific depletion of Foxp3+ Treg leads to inflamed and fibrosed tissue that hinders implantation	Teles et al. (6)
	Essential for implantation	Depletion of CD25+ cells impaired implantation	Shima et al. (3)
		Depletion of Foxp3+ cells hindered implantation	Teles et al. (6)

An unperturbed state of immune cell populations in the uterus during early pregnancy is of importance not only for implantation success but also for the quality of placentation. It is known that an inadequate immune response at the onset of pregnancy can result in shallow implantation and poor placentation, which can end in miscarriage, IUGR, or pre-eclampsia. Further, impaired growth *in utero* is implied in the development of metabolic diseases that affect the health of the offspring.

While much information is already available to foster our understanding of the complex circuit of immune cell interactions that supports early pregnancy success, more studies are necessary to understand this interplay in a holistic way. Approaches from system biology may contribute to this field in the future. There are still many open questions that we need to unravel in the future. Due to the increased number of pregnancies after assisted reproductive technologies, we need to understand how to interfere in order to support the generation of the optimal microenvironment for the blastocyst to attach. It is also very important to understand which fetal structures are crucial for the generation of tolerance. As pregnancies with fully foreign fetal antigens in egg donation procedures and surrogate mothers are possible, it is conceivable that fetal antigens are shed at a very early stage and contribute to the generation of tolerance. It is also theoretically possible that owing to the bidirectional chimerism fetal cells reach the maternal circulation of surrogate mothers in egg donation pregnancies, and this has an impact in later life, e.g., for the development of autoimmune diseases because of strong antigenicity of the fetus that is not only half foreign as it is in nature but also full foreign. These and many other open questions should be our challenge for the future and understanding the participation of immune cells at early pregnancy is fundamental for unraveling peri-implantation.

## Conflict of Interest Statement

The authors declare that the research was conducted in the absence of any commercial or financial relationships that could be construed as a potential conflict of interest.
